# Efflux Pumps and Porins Enhance Bacterial Tolerance to Phenolic Compounds by Inhibiting Hydroxyl Radical Generation

**DOI:** 10.3390/microorganisms13010202

**Published:** 2025-01-18

**Authors:** Xinyue Sui, Likun Guo, Zixian Bao, Mo Xian, Guang Zhao

**Affiliations:** 1 State Key Laboratory of Microbial Technology and Institute of Microbial Technology, Shandong University, Qingdao 266237, China; 2 CAS Key Lab of Biobased Materials, Qingdao Institute of Bioenergy and Bioprocess Technology, Chinese Academy of Sciences, Qingdao 266101, China

**Keywords:** phenolic compounds, efflux pumps, porins, toxicity, hydroxyl radical, phloroglucinol

## Abstract

Phenolic compounds are industrially versatile chemicals that have been successfully produced in microbial cell factories. Unfortunately, most phenolic compounds are highly toxic to cells in specific cellular environments or above a particular concentration because they form a complex with iron and promote hydroxyl radical production in Fenton reactions, resulting in the ferroptosis of cells. Here, we demonstrated that overexpression of efflux pumps and porins, including porins LamB and OmpN, and efflux pumps EmrAB, MdtABC, and SrpB, can enhance *Escherichia coli* phloroglucinol (PG) tolerance by inhibiting the generation of hydroxyl radicals. In addition, LamB and OmpN overexpression improved the bioproduction of PG. Furthermore, efflux pumps and porins can enhance bacterial tolerance to various phenolic compounds, including phenol, catechol, resorcinol, pyrogallol, and 2-naphthol. LamB and MdtABC confer a generalized tolerance to phenols. However, EmrAB, OmpN, and SrpB showed inconsistent effects of bacterial tolerance to different phenolic compounds. Our results will theoretically support the construction of phenolic compound-tolerant bacteria strains, which should be more efficient in the biosynthesis of phenols.

## 1. Introduction

As a broad class of fine or bulk chemicals, phenolic compounds clearly have wide applications in the industrial and consumer fields. For example, phenol is an important commodity chemical, conventionally used as a precursor for the synthesis of various plastics, synthetic fibers, phenolic resins, and synthetic rubber [1]. Phloroglucinol (PG) is an important aromatic platform compound, which itself is an excellent smooth muscle antispasmodic agent with good specificity [2]. It also has important applications in textile dyeing, artificial rainfall, and synthesizing an array of plastics [3].

Currently, phenol production is largely dependent on petrochemicals, but it often raises unsustainable, environmentally unfriendly, and economic and security concerns [1,4]. As a result, there is a strong interest in the use of microbial cell factories for the production of various phenolics from renewable biomass feedstocks [5,6,7]. Unfortunately, in specific cellular environments, most phenolic compounds have strong bactericidal properties even at a low concentration [6,7,8,9]; for example, the survival rate of *E. coli* BL21(DE3) was only 5% with the presence of 0.5 g/L PG in a minimal salt medium (MSM) [10], and when 5 mM phenol accumulated in the fermentation medium, the growth of the solvent-resistant strain *Pseudomonas putida* S12 (*P. putida* S12) was inhibited thereby limiting phenol production [11].

As reported, due to their hydrophobicity, phenolics can interact with the lipid bilayer of the cell membrane, disrupting membrane integrity and fluidity, affecting the function of the cell membrane, and releasing intracellular substances out of the cytoplasm, which can lead to impaired protein function and nutrient translocation [12,13]. Phenolic compounds with lower molecular masses exhibit greater toxicity compared to those with higher molecular masses, as compounds with a lower molecular weight could more rapidly enter the cell, enhancing the hindrance of glucose assimilation [14]. Additionally, phenolic compounds can increase intracellular reactive oxygen species (ROS) generation which causes oxidative stress, leading to denaturation and inactivation of proteins or enzymes as well as damage to DNA [13,15]. According to our previous reports, the mechanism of phenol-induced ROS production is elucidated, showing that complexes composed of phenols and iron were formed, promoting hydroxyl radical production in Fenton reactions, and, in turn, inducing ferroptosis-like cell death of *E. coli* [10].

In recent years, it has been reported that bacterial multidrug resistance is partially dependent on the active exocytosis of drugs; that is, exocytosis proteins or regulatory proteins are induced by antimicrobial drugs and expel the drugs, resulting in low intracellular drug concentration [16,17]. Multidrug efflux pumps are membrane proteins and can be categorized into six different families [18,19,20], including ATP-binding cassette (ABC) transporter, resistance nodulation division (RND), major facilitator superfamily (MFS), multidrug and toxic compound extrusion (MATE), small multidrug resistance (SMR), and proteobacterial antimicrobial compound efflux (PACE) [21]. The RND family has clinical significance because it confers endogenous resistance to Gram-negative bacteria and the associated infections are more difficult to treat [22,23]. The RND family consists of the inner membrane protein, the outer membrane channel protein, and the membrane-connected protein [24]. AcrAB-TolC, the most important efflux pump protein in *E. coli*, belongs to the RND family and non-selectively expels antibiotics from bacterial cells [23,25]. Upregulation of this efflux pump results in an elevated resistance against carbapenem, aminoglycosides, cephalosporins, and carbapenems [25]. In addition, MdtABC-TolC of *E. coli* and SrpABC of *P. putida* are also part of the RND family and are associated with resistance to antibiotics or organic solvents [26,27]. The MdtABC efflux system plays a dual function in bacteria, mediating multidrug resistance and maintaining bacterial iron homeostasis [26]. Heterologous expression of the efflux pump SrpABC in Escherichia coli improves tolerance to n-butanol [27]. The EmrAB-TolC belongs to MFS which is the largest and most diverse superfamily of secondary transporters, which not only play a crucial role in the transport of many substances but are also closely related to immunological issues such as viral invasion and bacterial resistance [20,28,29]. Porins constitute a significant proportion of outer membrane proteins within Enterobacteriaceae. It extends through the outer membrane, facilitating passive diffusion of small hydrophilic molecules into the periplasm, effectively reducing the intracellular concentration and resulting in antibiotic resistance [30,31]. There was a correlation between porin expression and the level of resistance to carbapenems within numerous Enterobacteriaceae strains exhibiting decreased susceptibility to carbapenems [32]. LamB could compensate for the absence of other nonspecific proteins to protect *Klebsiella pneumoniae* against cefoxitin [33]. Porin OmpN could enhance tetracycline resistance in *Vibrrio splendidus* [34] and replace the function of TolC in pumping drugs with the assistance of the protein AcrAB [35].

In this study, porins LamB and OmpN and efflux pumps EmrAB, MdtABC, and SrpB were identified to enhance *E. coli* PG tolerance by inhibiting the generation of hydroxyl radicals resulting from the PG–iron complex. LamB and OmpN overexpression improved the production of phloroglucinol. Furthermore, efflux pumps and porins could enhance bacterial tolerance to various phenolic compounds, like phenol, catechol, resorcinol, pyrogallol, and 2-naphthol.

## 2. Methods

### 2.1. Bacterial Strains and Growth Conditions

All bacterial strains used in this study are *E. coli*. The main flow of the experiment was carried out according to a study by Sui et al. and optimized for the specifics of the experiment [10]. Briefly, the construction of strains and plasmids was cultured with Luria–Bertani broth (Oxoid, Thermo Fisher Scientific, Waltham, MA, USA). All recombinant strains and plasmids used in this study are listed in Table 1. For phenol challenge and PG production, strains were cultured in a shake flask with MSM containing 9.8 g/L of K_2_HPO_4_ · 3H_2_O, 3.0 g/L of (NH_4_)_2_SO_4_, 2.1 g/L of citrate monohydrate, 0.3 g/L of ferric ammonium citrate, 0.24 g/L of MgSO_4_, 1 mL of trace element solution (3.7 g/L (NH_4_)_6_Mo_7_O_24_ · 4H_2_O, 2.9 g/L ZnSO_4_ · 7H_2_O, 24.7 g/L H_3_BO_3_, 2.5 g/L CuSO_4_ · 5H_2_O, and 15.8 g/L MnCl_2_ · 4H_2_O), and 20 g/L of glucose as a carbon source. When necessary, 100 μg/mL of ampicillin (Amp), 20 μg/mL of chloramphenicol (Cm), or 25 μg/mL of kanamycin (Kan) was added. If not specified, the reagents were purchased from Sinopharm Chemical Reagent Co., Ltd (Shanghai, China).

All primers used in this study are listed in Table S1. All primers were synthesized by Sangon Biotech (Shanghai, China), and the genes *srpA*, *srpB*, and *srpC* were synthesized by GenScrip Corporation (Nanjing, China). Plasmid constructions were performed by Clon Express Ultra One Cloning Kit (Vazyme Biotech, Nanjing, China) using *E. coil* DH5α as a host and confirmed by DNA sequencing (Sangon Biotech). *E. coil* BL21(DE3) Δ*tolC* was generated using the pCas/pRPS system [38].

For tolerance assay, strains carrying plasmid were cultured overnight and re-inoculated (1:50) into 100 mL MSM with 100 μg/mL Amp and induced by 0.1 mM IPTG at an OD_600_ of 0.8. When the strain was further grown to a late-exponential phase (OD_600_ of 2.5), the cells were collected to determine the colony-forming unit (CFU); meanwhile, phenolic compounds were added in MSM, and the cells were further grown. Specifically, 1.3 g/L PG (Aladdin, Shanghai, China) was used for 4 h. In addition, 3 g/L phenol (Macklin, Shanghai, China), 3.2 g/L catechol (Aladdin, Shanghai, China), 3 g/L resorcinol (Aladdin, Shanghai, China), 3.5 g/L pyrogallol (Aladdin, Shanghai, China), and 0.27 g/L 2-naphthol (Aladdin, Shanghai, China) were used for 5 h. Then, the cells were collected to determine CFU, intracellular iron, and hydroxyl radical concentrations. Survival = (CFU with phenolic compound challenge/CFU without phenolic compound challenge). The intracellular iron and hydroxyl radical concentrations were determined using the Iron Colorimetric Assay Kit (Applygen Technologies, Beijing, China) and Hydroxyphenyl fluorescein (Shanghai Maokang Biotechnology, Shanghai, China), respectively.

### 2.2. Protein Expression and Gel Electrophoresis Analysis

Strains were cultured in 50 mL fresh MSM. Then, 0.1 mM IPTG was added when strains were grown to an OD_600_ of 0.8 and further grown to an OD_600_ of 2.5. Cells were collected and disrupted by high pressure and centrifuged to separate the supernatant and precipitate. Then, proteins were analyzed by 12% SDS-PAGE.

### 2.3. Biosynthesis of PG

PG biosynthesis was performed in 250 mL shake flasks containing 50 mL of MSM with 20 μg/mL Cm. After incubation to OD_600_ 0.8 at 37 °C, 0.1 mM IPTG was added and then the temperature was decreased to 30 °C for further culture of 24 h. PG concentration was detected using the colorimetric reaction between PG and cinnamaldehyde at 446 nm.

## 3. Results

### 3.1. Identification of Efflux Pumps and Porin Gene Essential to PG Tolerance

**Figure 1:**
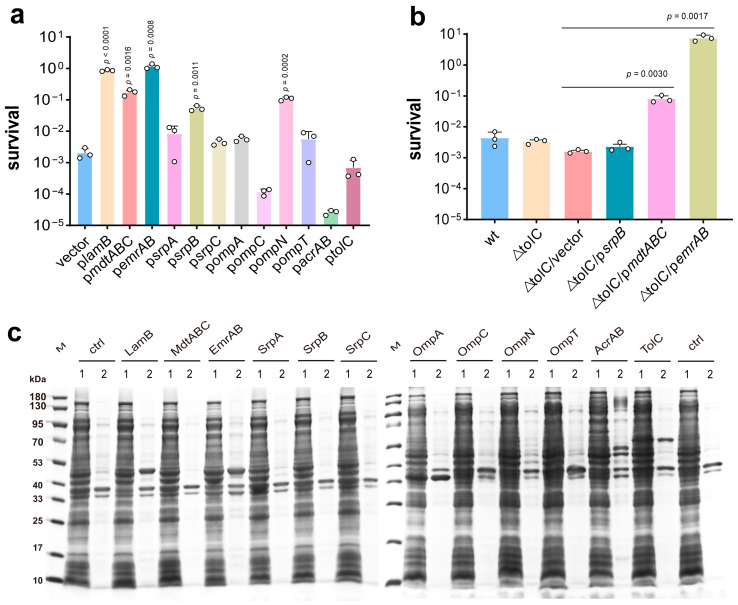
Identification of efflux pumps and porins related to phloroglucinol (PG) tolerance. (**a**) Tolerance of *E. coli* BL21(DE3) strain carrying empty vector or membrane protein overexpression vector after treatment with PG at 1.3 g/L for 4 h. The circle represents a data (*n* = 3 biological independent samples). (**b**) Confirmation of the role of TolC, EmrAB, MdtABC, and SrpB in PG tolerance by knockout *tolC*, and EmrAB, MdtABC, and SrpB overexpression in *tolC* knockout strain. The circle represents a data (*n* = 3 biological independent samples). (**c**) The protein of different strains from samples at an OD_600_ of 2.5. A total of 0.1 mM IPTG was added to induce proteins when OD_600_ was 0.8. Lane M, pre-stained protein molecular weight marker (kDa); lane 1, soluble proteins in the supernatant; lane 2, insoluble proteins in precipitation. LamB, 49.9kDa; MdtA, 44.5 kDa; MdtB, 112.1 kDa; MdtC, 111.0 kDa; EmrA, 42.7kDa; EmrB, 55.6kDa; SrpA, 41.4 kDa; SrpB, 114.1 kDa; SrpC, 51.4 kDa; OmpA, 37.2kDa; OmpC, 34.4 kDa; OmpN, 41.2 kDa; OmpT, 35.6 kDa; AcrA, 42.2 kDa; AcrB, 113.6 kDa; TolC, 53.7 kDa.

In our previous experiments, it was proven that the generation of hydroxyl radicals (HO·) promoted by the PG–iron complex was the main factor of PG toxicity to *E. coli* BL21(DE3) [10]. Based on this, reducing the intracellular PG concentration is supposed to enhance cell tolerance. To test whether efflux pumps and porins affect PG tolerance, genes encoding nine efflux pumps and porins of *E. coli* and three proteins of *P. putida* were cloned into plasmid vector pTrcHis2B and overexpressed in the BL21(DE3) strain, respectively, including *acrAB* operon, *tolC*, *mdtABC* operon, *emrAB* operon, *lamB*, *ompN*, *ompA*, and *ompC* from *E. coli* BL21(DE3), *ompT* from *E. coli* W3110, and *srpA*, *srpB*, and *srpC* from *P. putida* S12. The strains were cultured to the late-exponential phase (OD_600_ 2.5) in MSM and then treated with 1.3 g/L PG for 4 h. As shown in Figure 1a, almost all strains carrying empty vectors were killed by PG, and only 0.2% cells survived. The PG tolerance of SrpA, SrpC, OmpA, OmpT, and TolC overexpression strains was similar to strains carrying empty vectors, whereas overexpression of MdtABC, EmrAB, LamB, OmpN, and SrpB could improve the host tolerance against PG, especially LamB and EmrAB, with survival rates of 80% and 115.3%, respectively. Overexpression of MdtABC, OmpN, and SrpB also restored bacterial survival to 17.3%, 10.9%, and 5%, respectively. In addition, p*ompC* and p*acrAB* strains became much more susceptible to PG than the wild-type strain, and the growth of the p*acrAB* strain was significantly inhibited even in the absence of PG, probably because the excessive AcrAB level affected normal growth and metabolism of this strain. SrpABC was similar to the AcrAB-TolC efflux pump. In general, EmrAB, MdtABC, and AcrAB in *E. coli* need to be together with TolC to function as efflux pumps. To further verify that TolC in the efflux pump was not associated with PG tolerance, the *tolC* gene was knocked and tested for PG toxicity. The survival rate of the Δ*tolC* strain was similar to that of wild *E. coli* BL21(DE3) (Figure 1b). Following this, the function of EmrAB, MdtABC, and SrpB was confirmed by overexpression in the *tolC* knockout strain (Figure 1b). Both EmrAB and MdtABC increased PG tolerance regardless of the presence or absence of the TolC protein (Figure 1a,b). SrpB was promoted more significantly in the presence of TolC (Figure 1a,b). The above results indicated that EmrAB and MdtABC improved PG tolerance in *E. coli* independently of TolC, and SrpB probably required TolC for its role in *E. coli*. Overall, five membrane proteins (MdtABC, OmpN, SrpB, LamB, and EmrAB) proved to be instrumental in improving the PG tolerance of *E. coli*.

Apart from that, the protein expression level was further confirmed using SDS-PAGE and Coomassie blue staining (Figure 1c). Despite the fact that overexpression of LamB, EmrAB, MdtABC, OmpN, and SrpB improved the bacterial PG tolerance, LamB and EmrAB proteins were mainly present in inclusion bodies, and no obvious band of MdtABC, OmpN, and SrpB was observed in either the cell lysate supernatant or the precipitation (Figure 1c), suggesting that only a small amount of efflux pumps and porins were needed to play the role of material efflux and improve PG tolerance. Although TolC, OmpA, and SrpA were partially soluble expressed, they did not promote bacterial survival under PG stress, implying that they are not related to the PG tolerance of *E. coli*. In addition, OmpC, OmpT, and AcrAB were misfolded or aggregated to form inclusion bodies, and no SrpC band was observed because of the low expression.

### 3.2. Efflux Pumps and Porins Inhibit the Generation of Hydroxyl Radical

**Figure 2:**
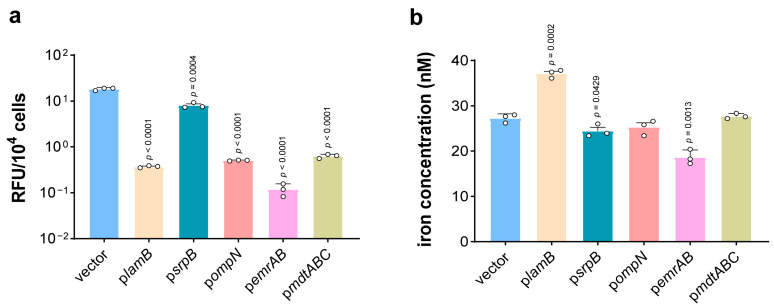
Efflux pumps and porins inhibited generation of hydroxy radicals (HO·). (**a**) Intracellular HO· concentration determined of *E. coli* BL21(DE3) strain carrying empty vector or membrane protein overexpression vector grown in MSM after challenge of 1.3 g/L PG for 4 h. The circle represents a data (*n* = 3 biological independent samples). (**b**) Intracellular iron concentration in *E. coli* BL21(DE3) strain carrying empty vector or membrane protein overexpression vector after challenge of 1.3 g/L PG for 30 min. The circle represents a data (*n* = 3 biological independent samples).

According to our previous report, the PG–iron complex promotes the generation of HO·. In order to verify whether overexpression of the above five proteins could inhibit the intracellular Fenton reaction, the intracellular levels of HO· were examined with 1.3 g/L PG for 4 h. As we expected, the higher tolerance the strain has, the lower the intracellular HO· level that was detected. Strains carrying empty vectors presented a HO· concentration of 153 times and 49 times as high as p*emrAB* and p*lamB*, which significantly restored the viability of the *E. coli* BL21(DE3) strain (Figure 2a). Next, p*ompN* and p*mdtABC* were found to be at least 29 times lower than the pVector strain. In addition, the HO· content of p*srpB* was only about 56% lower than that of the control strain, which slightly affected PG susceptibility. All these results demonstrated that efflux pumps and porins protect *E. coli* from PG toxicity as they limit the potential for PG–iron complex-dependent HO· formation.

### 3.3. Comparison of Intracellular Iron Levels

Iron is known to be necessary for the toxicity of phenolic compounds [10]. Next, it was determined whether the intracellular iron levels of membrane protein overexpression strains changed. As expected, p*emrAB* had a lower iron concentration than the control strain (p*emrAB* 18.65 ± 1.77 and pVector 27.28 ± 1.08). Surprisingly, intracellular iron content was significantly increased in the strain overexpressing LamB, which was 36% higher than the pVector strain (Figure 2b). LamB has been shown to compensate for the absence of other nonspecific proteins to improve strain tolerance [33]. We speculate that LamB avoids ferroptosis-like death by excluding PG from the body rather than iron. RND-type MdtABC is a drug efflux pump that can export antibiotics [39]. It has been shown that MdtABC and AcrD cooperate with AcrB to excrete enterobactin (a siderophore) from the cytosol to the extracellular space [26]. But in this study, there was no difference in iron concentration (27.71 ± 0.64) compared to the control strain (Figure 2b). Similarly, p*ompN* and p*srpB* strains showed no significant changes in iron ion levels (Figure 2b). According to the results of the intracellular levels of HO· and iron, the enhancement of PG resistance should be due to the reduction in intracellular PG concentration.

### 3.4. Porin Overexpression Benefits Biosynthesis of PG

**Figure 3:**
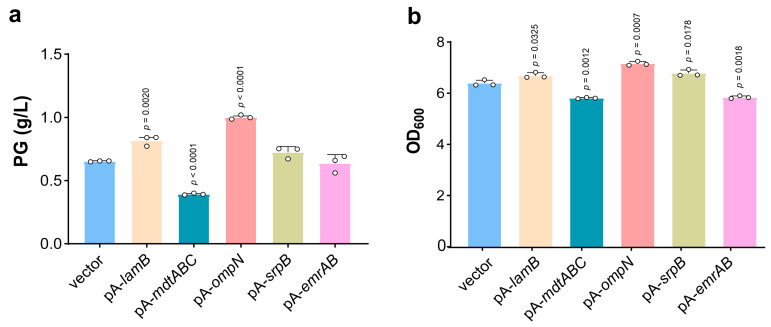
Biosynthesis of PG. (**a**) PG production of membrane protein overexpression strains. The circle represents a data (*n* = 3 biological independent samples). (**b**) Growth of membrane protein overexpression strains. The circle represents a data (*n* = 3 biological independent samples).

In recent years, the synthesis of phenolic compounds by microorganisms has become a research frontier in the world. However, *E. coli* suffers from the toxicity of PG with growth inhibition, limiting the maximum titer of PG accumulated in the medium [40]. To test the influence of our screened efflux pumps and porins on PG biosynthesis, five proteins that enhance PG tolerance were cloned into pA-*phlD*/*marA/acc* carrying PG biosynthetic pathways and then introduced into *E. coli* BL21(DE3). In shake flask cultivation, the pA-*ompN* strain produced 1.00 ± 0.02 g/L PG and the pA-*lamB* strain produced 0.82 ± 0.04 g/L PG, respectively, which is 53.85% and 26.15% higher than that of the wild-type strain (0.65 ± 0.00 g/L) (Figure 3a). And the cell density (OD_600_) of pA-*ompN* was increased to 7.16 ± 0.09, while the control strain was 6.39 ± 0.14 (Figure 3b). Although MdtABC, EmrAB, and SrpB enhanced the survival rate in the presence of PG (Figure 1a), the PG production was instead reduced (Figure 3a), probably because the exogenous protein expression increased the metabolic burden of the strain or affected the normal metabolism of the strains. This demonstrated that overexpression of porins improves cellular tolerance to phenolics and benefits phenol-related biochemical processes.

### 3.5. Efflux Pumps and Porins Improve Bacterial Tolerance to Diverse Phenolic Compounds

**Figure 4:**
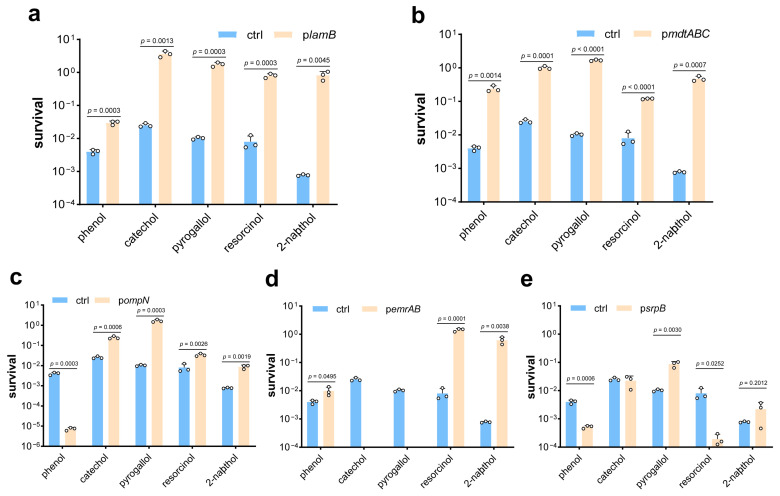
Efflux pumps and porins improve tolerance to diverse phenolic compounds. Tolerance of *E. coli* BL21(DE3) strain carrying empty vector and p*lamB* (**a**), p*mdtABC* (**b**), p*ompN* (**c**), p*emrAB* (**d**), or p*srpB* (**e**) upon exposure to different phenolic compounds. The circle represents a data (*n* = 3 biological independent samples).

Some other phenolic compounds including phenol [11,41], resorcinol [42], catechol [7,43], pyrogallol [9,44], and 2-naphthol [45] were produced or degraded by microorganisms. Therefore, they were tested for cytotoxicity to confirm the abilities of efflux pumps or porins to improve tolerance. Figure 4a showed that the viability of the p*lamB* strain was enhanced dramatically in the presence of all five different phenolic compounds. Similar results were also observed in the strain p*mdtABC* (Figure 4b). This suggested that LamB and MdtABC confer a generalized tolerance to phenols. However, overexpression of other proteins showed inconsistent effects on bacterial tolerance to various phenolic compounds. The p*ompN* strain showed a significant reduction in sensitivity to the tested compounds except for phenol (Figure 4c). In regard to the p*emrAB* strain, all cells exposed to catechol and pyrogallol were killed, while its tolerance to resorcinol and 2-naphthol was highly improved (Figure 4d). Interestingly, though SrpB is derived from solvent-resistant *P. putida*, the p*srpB* strain was less resistant to phenol and resorcinol (Figure 4e). Substrate specificity of proteins may be responsible for differences in the tolerance of strains to different phenolics.

## 4. Discussion

Phenolic compounds play an important role in the medicine, food, cosmetics, textiles, and chemical industries. In recent years, the synthesis of phenolic compounds by microorganisms has attracted more attention. However, the toxic effects of phenolics on bacteria are a factor in its yield limitation. According to the results shown above, we discovered that efflux pumps and porins significantly restored the viability of strains with the presence of phenolic compounds. Moreover, LamB and MdtABC had a generalized tolerance to phenols, such as PG, phenol, catechol, resorcinol, pyrogallol, and 2-naphthol (Figure 1a and Figure 4). LamB could compensate for the absence of other nonspecific proteins to protect strains from harm [33]. This may explain why it can enhance phenol tolerance. MdtABC requires TolC for its efflux function, which is associated with resistance to antibiotics, bile salt derivatives, and SDS [39]. OmpN is a nonspecific porin and not highly expressed under typical laboratory growth conditions. When overexpressed, it operates similarly to OmpC. But due to the different substrate specificity between the two proteins [46], the PG tolerance of p*ompN* was significantly enhanced, whereas p*ompC* was the same as that of controls (Figure 1a). The *srpB* gene is derived from solvent-resistant *P. putida* and improves *n*-butanol tolerance in *E. coil* [27], but in our study, the p*srpB* strain was less resistant to phenol and resorcinol, and the tolerance to PG just slightly improved (Figure 1a and Figure 4). AcrAB-TolC is the most important efflux pump in bacteria since it is not substrate specific [23], but its response to PG was ineffective, and the p*acrAB* strain almost stopped growing even in the absence of PG. To our knowledge, this is the first report of MdtABC, OmpN, and EmrAB being associated with bacterial tolerance to phenolic compounds. The phenolic compounds used in this study are basic molecular structures, each comprising just one or two benzene rings, capable of triggering ferroptosis in a variety of organisms. In contrast, polyphenols are a category of natural substances prevalent in advanced plant species and are usually composed of more benzene ring structures and diverse functional groups, exhibit antioxidant properties, and are effective in inhibiting ferroptosis [47]. The variance in their biological activities is likely attributable to the distinct molecular structures between simple phenols and polyphenolic compounds.

**Figure 5:**
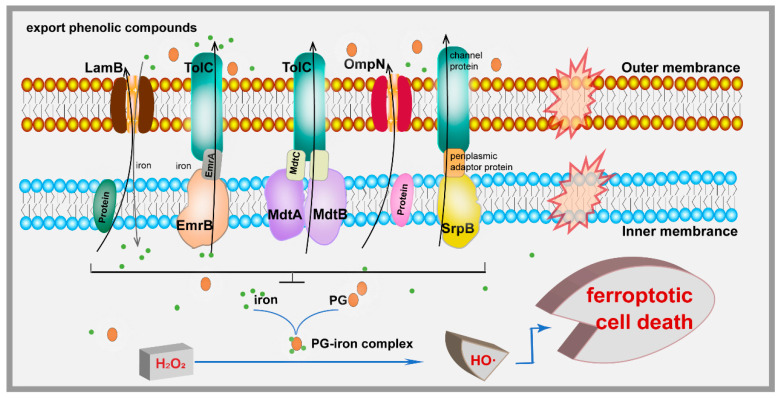
Model illustrating the mechanism of the efflux pumps and porins that increased phenolic tolerance.

The PG–iron complex promotes the generation of HO·, causing ferroptosis-like cell death. In this study, efflux pumps and porins were demonstrated to reduce intracellular HO· accumulation, especially LamB and EmrAB, being 153 times and 49 times as low as the wild strain, respectively, and effectively mitigating ferroptosis-like death of *E. coli* (Figure 2a and Figure 5). In addition, our research has great application potential in the biosynthesis of phenolic compounds. The pA-*ompN* strain produced a PG titer that was 53.85% higher than that of the wild-type strain (Figure 3a). The enhancement of bacterial tolerance to phenolic compounds can theoretically prolong the fermentation process, but the fermentation process can be affected by a variety of factors, such as enzyme activity, plasmid stability, the medium, and culture conditions. It has been shown that an enhanced tolerance to exogenous solvents does not inherently result in a higher yield of that solvent [48]. Future studies can combine multiple factors to boost the production of phenolics.

In conclusion, this study provides an effective method for optimizing phenolic tolerance in *E. coli* and the development of PG production. Our study will provide some theoretical basis for the construction of phenol-tolerant strains and the biosynthesis of phenols.

## 5. Conclusions

This study investigated the effects of various efflux pumps and porins on bacterial phenolic compound tolerance. The results indicated that five proteins, LamB, OmpN, EmrAB, MdtABC, and SrpB, significantly improved *E. coli* PG resistance. And the higher tolerance the strain has, the lower intracellular the HO· level that was detected. The enhancement of tolerance is due to the efflux pumps and porins inhibiting the formation of HO· induced by the PG–iron complex. Furthermore, the PG biosynthesis experiment showed that overexpression of porins OmpN and LamB improved PG production, which was beneficial for phenol-related biochemical processes. In addition, efflux pumps and porins could significantly restore the viability of strains with the presence of various phenolic compounds, including phenol, resorcinol, catechol, pyrogallol, and 2-naphthol. These findings will provide a theoretical foundation for the development of bacterial strains with enhanced tolerance to phenolic compounds.

## Figures and Tables

**Table 1:** Bacterial strains and plasmids used in this study.

Strain or Plasmid	Description	Source
*E. coil* DH5α	F^−^ *supE*44 Δ*lacU*169 (*ϕ*80 *lacZ* Δ*M15*) *hsdR*17 *recA*1 *endA1 gyrA*96 *thi*-1 *relA*1	Lab collection
*E. coil* BL21(DE3)	F^−^ *ompT gal dcm lon hsdSB* (rB^−^ mB^−^) λ(DE3)	Lab collection
*E. coil* W3110	F- λ- rph-1 INV (rrnD, rrnE)	Lab collection
Q3595	*E. coil* BL21(DE3)/pA-*phlD/marA/acc*	[36]
Q3861	*E. coil* BL21(DE3)/pTrcHis2B	This study
Q5660	*E. coil* BL21(DE3)/pTRC-*tolC*	This study
Q5661	*E. coil* BL21(DE3)/pTRC-*ompA*	This study
Q5662	*E. coil* BL21(DE3)/pTRC-*ompN*	This study
Q5663	*E. coil* BL21(DE3)/pTRC-*lamB*	This study
Q5664	*E. coil* BL21(DE3)/pTRC-*acrAB*	This study
Q5665	*E. coil* BL21(DE3)/pTRC-*ompC*	This study
Q5666	*E. coil* BL21(DE3)/pTRC-*emrAB*	This study
Q5667	*E. coil* BL21(DE3)/pTRC-*mdtABC*	This study
Q6285	*E. coil* BL21(DE3)/pTRC-*ompT*	This study
Q6286	*E. coil* BL21(DE3)/pTRC-*srpA*	This study
Q6287	*E. coil* BL21(DE3)/pTRC-*srpB*	This study
Q6288	*E. coil* BL21(DE3)/pTRC-*srpC*	This study
Q6329	*E. coil* BL21(DE3)/pA-*lamB*	This study
Q6330	*E. coil* BL21(DE3)/pA-*ompN*	This study
Q6331	*E. coil* BL21(DE3)/pA-*srpB*	This study
Q6332	*E. coil* BL21(DE3)/pA-*mdtABC*	This study
Q6333	*E. coil* BL21(DE3)/pA-*emrAB*	This study
Q6424	*E. coil* BL21(DE3) Δ*tolC*	This study
Q6425	*E. coil* BL21(DE3) Δ*tolC/*pTrcHis2B	This study
Q6426	*E. coil* BL21(DE3) Δ*tolC/*pTRC-*emrAB*	This study
Q6427	*E. coil* BL21(DE3) Δ*tolC/*pTRC-*mdtABC*	This study
Q6428	*E. coil* BL21(DE3) Δ*tolC/*pTRC-*srpB*	This study
Plasmids		
pTrcHis2B	rep_pBR322_ Amp^R^ *lacI*^q^ P*_trc_*	Invitrogen
pA-p*hlD/marA/acc*	rep_p15A_ Cm^R^ *lacI* P_T7_-*phlD-marA-accADBC*	[36]
pCas	rep_pSC101_^Ts^ Kan^R^ P_cas_-*cas9* P*_araB_*-Red *lacI*^Q^ P_trc_-sgRNA_pMB1_	MolecularCloud: MC0000011 [37]
pPaper-Δ*tolC*	rep_p15A_ Cm^R^ P_lacIQ_-sgRNA-Tet^R^ P_J23119_-sgRNA Δ*tolC*	This study
pTRC-*lamB*	rep_pBR322_ Ap^R^ *lacI*^q^ P*_trc_-lamB*	This study
pTRC-*ompN*	rep_pBR322_ Ap^R^ *lacI*^q^ P*_trc_-ompN*	This study
pTRC-*tolC*	rep_pBR322_ Ap^R^ *lacI*^q^ P*_trc_-tolC*	This study
pTRC-*srpA*	rep_pBR322_ Ap^R^ *lacI*^q^ P*_trc_-srpA*	This study
pTRC-*srpB*	rep_pBR322_ Ap^R^ *lacI*^q^ P*_trc_-srpB*	This study
pTRC-*srpC*	rep_pBR322_ Ap^R^ *lacI*^q^ P*_trc_-emrAB*	This study
pTRC-*emrAB*	rep_pBR322_ Ap^R^ *lacI*^q^ P*_trc_-emrAB*	This study
pTRC-*mdtABC*	rep_pBR322_ Ap^R^ *lacI*^q^ P*_trc_-mdtABC*	This study
pTRC-*acrAB*	rep_pBR322_ Ap^R^ *lacI*^q^ P*_trc_-acrAB*	This study
pTRC-*ompA*	rep_pBR322_ Ap^R^ *lacI*^q^ P*_trc_-ompA*	This study
pTRC-*ompC*	rep_pBR322_ Ap^R^ *lacI*^q^ P*_trc_-ompC*	This study
pTRC-*ompN*	rep_pBR322_ Ap^R^ *lacI*^q^ P*_trc_-ompN*	This study
pTRC-*ompT*	rep_pBR322_ Ap^R^ *lacI*^q^ P*_trc_-ompT*	This study
pA-*lamB*	rep_p15A_ Cm^R^ *lacI* P_T7_-*phlD-marA-accADBC-*P*_trc_-lamB*	This study
pA-*ompN*	rep_p15A_ Cm^R^ *lacI* P_T7_-*phlD-marA-accADBC-*P*_trc_-ompN*	This study
pA-*srpB*	rep_p15A_ Cm^R^ *lacI* P_T7_-*phlD-marA-accADBC-*P*_trc_-srpB*	This study
pA-*mdtABC*	rep_p15A_ Cm^R^ *lacI* P_T7_-*phlD-marA-accADBC-*P*_trc_-mdtABC*	This study
pA-*emrAB*	rep_p15A_ Cm^R^ *lacI* P_T7_-*phlD-marA-accADBC-*P*_trc_-emrAB*	This study

## Data Availability

The original contributions presented in this study are included in the article/Supplementary Materials. Further inquiries can be directed to the corresponding author.

## Supplementary Materials

The following supporting information can be downloaded at https://www.mdpi.com/article/10.3390/microorganisms13010202/s1: Table S1: Primers used in this study.
