# Supplementary material for: Efflux Pumps and Porins Enhance Bacterial Tolerance to Phenolic Compounds by Inhibiting Hydroxyl Radical Generation
Source: Microorganisms. 2025 Jan 18;13(1):202. doi: 10.3390/microorganisms13010202 (PMC11767505; doi:10.3390/microorganisms13010202)
Supplement: Supplementary file 1 [file microorganisms-13-00202-s001.zip › microorganisms-3418694-supplementary.pdf]

Table S1. Primers used in this study

primer	sequence
**Application of backbone of pTrcHis2B**	
pTrc-F	TGGTACCATATGGGAATTCGAAG
pTrc-R	AGATCTCGAGCTCGGATCCATG
**pTRC-*lamB***	
guo-lamBF	GATCCGAGCTCGAGATCTATGATGATTACTCTGCGCAAACTTCCTC
guo-lamBR	AATTCCCATATGGTACCATTACCACCAGATTTCCATCTGGGCAC
**pTRC-*ompN***	
guo-ompNF	GATCCGAGCTCGAGATCTATGAAAAGCAAAGTACTGGCAC
guo-ompNR	AATTCCCATATGGTACCATTAGAACTGATAAACCAGACCTAAAGC
**pTRC-*tolC***	
guo-tolCF	GATCCGAGCTCGAGATCTATGAAGAAATTGCTCCCCATTC
guo-tolCR	AATTCCCATATGGTACCATCAGTTACGGAAAGGGTTATG
**pTRC-*acrAB***	
guo-acrABF	GATCCGAGCTCGAGATCTATGAACAAAAACAGAGGGTTTACGCCTC
guo-acrABR	AATTCCCATATGGTACCATCAATGATGATCGACAGTATGGCTG
**pTRC-*ompA***	
guo-ompAF	GATCCGAGCTCGAGATCTATGAAAAAGACAGCTATCGCGATTG
guo-ompAR	AATTCCCATATGGTACCATTAAGCCTGCGGCTGAGTTAC
**pTRC-**	

emrAB	
guo-emrABF	GATCCGAGCTCGAGATCTATGAGCGCAAATGCGGAGACTC
guo-emrABR	AATTCCCATATGGTACCATTAGTGCGCACCGCCTCC
**pTRC-*mdtABC***	
guo-mdtABCF	GATCCGAGCTCGAGATCTATGAAAGGCAGTTATAAATCCCGTTG
guo-mdtABCR	AATTCCCATATGGTACCATTACTCGGTTACCGTTTGTTTAGG
**pTRC-*ompC***	
guo-ompCF	GATCCGAGCTCGAGATCTATGCGTCTTGGCTTCAAAGGTG
guo-ompCR	AATTCCCATATGGTACCATTAGAACTGGTAAACCAGACCCAG
**pTRC-*ompT***	
guo-ompTF	GATCCGAGCTCGAGATCTATGCGGGCGAAACTTCTG
guo-ompTR	AATTCCCATATGGTACCATTAAAATGTGTACTTAAGACCAGCAG
**pTRC-*srpA***	
guo-srpAF	GATCCGAGCTCGAGATCTATGCGACAAATAAGATCACCCAG
guo-srpAR	AATTCCCATATGGTACCATTAATTCGCGCTCGCCTC
**pTRC-*srpB***	
guo-srpBF	GATCCGAGCTCGAGATCTATGTCAAGATTCTTTATTGATAGGCCCA
guo-srpBR	AATTCCCATATGGTACCATTACACCTCATGGGTCACTTCCTTC
**pTRC-*srpC***	

guo-srpCF	GATCCGAGCTCGAGATCTATGAAATTCAAGTCACTACCCATGTTTG
guo-srpCR	AATTCCCATATGGTACCATTAGTTTTGGCTAACGCTCCAAC
**pACYC-*marA*-*accADBC*-*phlD*-P$_{trc}$-gene**	
gene-F	AACGGTTCTGGCAAATATTCTG
gene-R	AGCTTCGAATTCCCATATGGTAC
pA-F2	TGGTAATGGTACCATATGGGAATTCGAAGCTCTGCGAAATTTGAACGCCAGCAC
pA-R2	TCATTTCAGAATATTTGCCAGAACCGTTCAGCGGTTTCTTTACCAGACTCGAG
**pPaper-Δ*tolC***	
BtolCgF	CCTAGGTATAATACTAGTCGGTAACTACTATCCGGAACGTTTTAGAGCTAGAAATAGCA
gRNAR5	GTAATAGATCTAAGCTTCTGC
H-BtolCHLF	GAAGCTTAGATCTATTACTATCTTCGTTTTCATCAGCCAGACC
BtolCHLR	TAAGGCGTGCTTGCTGATAAACTTG
BtolCHRF	ATCAGCAAGCACGCCTTATGCGACCACCACGTTGTACAAC
H-BtolCHRR	TCGAGTAGGGATAACAGGAGAAACTCCCGCTGGATTGC
Pre_BtolCF	TTGATGATGCAGCTGCAGCCATTG
Aft_BtolCR	TGGCTGGGTTTTTCGAGGAGAAC
